# 

**Published:** 1880-04

**Authors:** 


*CONTENTS.*

| | PAGE |
|---|---|
| Healthy Bread | 95 |
| Sores | 98 |
| Heart Disease | 99 |
| Sleeping Together | 100 |
| A Surfeit | 102 |
| Spring Diseases | 102 |
| Rheumatism | 103 |
| Cure for Drunkenness | 104 |
| The Human Teeth | 105 |
| Checking Perspiration | 106 |
| Death's Weapons | 107 |
| Reading | 108 |
| Food Adulterations | 109 |
| Spring Diseases | 110 |
| Child Murder | 111 |
| Fever and Ague | 111 |
| Cholera Certainties | 116 |
| Reading | 120 |
| Wakefulness | 121 |
| Carelessness | 122 |
| Constipation | 123 |
| Biliousness | 124 |
| Bowel Complaints of Children | 125 |

